# Effects of whole body vibration exercise on neuromuscular function for individuals with knee osteoarthritis: study protocol for a randomized controlled trial

**DOI:** 10.1186/s13063-017-2170-6

**Published:** 2017-09-20

**Authors:** Zhangqi Lai, Xueqiang Wang, Seullee Lee, Xihe Hou, Lin Wang

**Affiliations:** 1 School of Kinesiology, Shanghai University of Sport, Shanghai, China; 2 Key Laboratory of Exercise and Health Sciences, Ministry of Education, Shanghai University of Sport, Shanghai, China; 3 Sport Coaching School, Shanghai University of Sport, Shanghai, China

**Keywords:** Neuromuscular function, Randomized controlled trial, Whole body vibration, Strength training, Knee osteoarthritis

## Abstract

**Background:**

Knee osteoarthritis (KOA) is a leading cause of public disability. Neuromuscular function contributes to the development and/or progression of KOA. Whole body vibration (WBV) exercise improve the neuromuscular function of patients with neurological disorders and even that of older patients with limited exercise options. Therefore, WBV exercise may offer an efficient and alternative treatment for individuals with KOA. However, the effects of WBV training on the neuromuscular function of individuals with KOA remain unclear. Therefore, this study attempts to investigate the effect of a 12-week WBV exercise on the neuromuscular function of individuals with KOA.

**Methods/design:**

We will conduct a prospective, single-blind randomized controlled trial on 180 KOA patients. Participants will be randomly assigned to the WBV exercise, lower extremity resistance training, and health education groups. The WBV exercise group will participate in a 12-week WBV training. The lower extremity resistance training group will undergo a 12-week lower extremity resistance training of both lower limbs. The control group will receive health education for 12 weeks. After the intervention, the participants will be followed up for 3 months with no active intervention. Primary outcome measures will include anthropometric measurements, gait analysis during walking and stair climbing, muscle strength test of the knee and ankle, proprioception test of the knee and ankle, and neuromuscular response of the leg muscles. Secondary outcome measures will include self-reported pain and physical functional capacity, and physical performance measures. Furthermore, adverse events will be recorded and analyzed. If any participant withdraws from the trial, intention-to-treat analysis will be performed.

**Discussion:**

Important features of this trial mainly include intervention setting, outcome measure selection, and study duration. This study is intended for estimating the effect of WBV intervention on neuromuscular control outcomes. Study results may provide evidence to support the beneficial effects of WBV exercise on the physical performance and neuromuscular control of individuals with KOA to fill the research gap on the efficacy of WBV.

**Trial registration:**

Chinese Clinical Trial Registry, ID: ChiCTR-IOR-16009234. Registered on 21 September 2016.

**Electronic supplementary material:**

The online version of this article (doi:10.1186/s13063-017-2170-6) contains supplementary material, which is available to authorized users.

## Background

Osteoarthritis (OA) is a major cause of disability in the aging population; this condition has increased in prevalence in recent years, and its consequences significantly affect society [1, 2]. In the United States in 2005, OA affected 13.9% of adults aged 25 years and older and 33.6% (about 12.4 million) of those aged 65 years and older [3]. In some regions of China, the prevalence of knee osteoarthritis (KOA) is 42.8% in women and 21.5% in men aged 60 years and older [4]. A report from the World Health Organization indicated that 80% of OA patients older than 60 years experience limited movements and 25% cannot perform major daily activities [5]. Hip and knee OA is a leading cause of global disability; this condition was ranked the 11th highest contributor to global disability and the 38th highest in disability-adjusted life years among 291 conditions [6].

About 13% of women and 10% of men aged 60 years and older suffer from symptomatic KOA [2]. Individuals with KOA often report joint pains, stiffness, and impaired body functions such as muscle strength, proprioception, and joint stability [7]. No cure for KOA currently exists. The management of KOA is broadly divided into nonpharmacological, pharmacological, and surgical treatments [8]. The optimal management of patients with KOA requires a combination of pharmacological and nonpharmacological treatments, and even sometimes surgical treatment [9]. The American College of Rheumatology recommends that aerobic and/or resistance land-based exercise, aquatic exercise, weight loss, Tai Chi, traditional Chinese acupuncture, patient education, and using wedged insoles are effective nonpharmacological treatments for patients with KOA [9].

Exercise and physical therapies are recommended for the nonpharmacological management of KOA; these treatments may be important alternatives for bridging the gap between the disease onset and a final operative intervention [10, 11]. Recent studies have considered whole body vibration (WBV) exercise as an efficient and alternative treatment for individuals with KOA [12, 13]. WBV exercise is easy and safe to perform. In WBV exercise, vibration signals are delivered through a vibratory platform or chair to expose a larger part of the body to the stimulation [14]. WBV exercise provides an amplitude of displacement (0.7–14 mm) and a mechanical oscillation of a specific frequency (0.5–80 Hz) [15, 16]. WBV exercise reduces pain and improves physical functions in patients with KOA; hence, it is suggested that it be included in rehabilitation programs [12, 13].

Neuromuscular function is the ability of the nervous system to produce muscular activities and maintain body movements through integration of the afferent signals from peripheral neurons and control of the efferent signals [17]. Neuromuscular function contributes to the development and/or progression of KOA [18]. Individuals with KOA exhibit impaired proprioceptive function of the affected joint compared with age-matched controls [19]. A narrative review reported that the impairment of proprioception might play a vital but undefined role in KOA, and the effect of exercise therapy on proprioceptive accuracy in patients with KOA is required [19]. The quadriceps femoris muscle is significantly impaired in patients with KOA; both activation deficit and atrophy contribute to quadriceps weakness [20]. Muscle impairments in patients with KOA also involve hamstrings and hip muscles [20]. Therefore, improvement of the proprioceptive function and quadriceps strength is important for KOA management. Various studies have reported that WBV exercise improves the neuromuscular function of healthy individuals, patients with neurological disorders, and even older patients with limited exercise options [21, 22]. WBV exercise improves the muscle strength, power, joint proprioception, balance, and flexibility of sedentary and older individuals [23]. In particular, WBV exercise improves quadriceps strength [24, 25]. During WBV exercise, vibrations are transmitted to the body and stimulate the primary ending of the muscle spindles, thereby activating σ-motor neurons, which cause muscle contractions similar to the tonic vibration reflex [26]. Thus, WBV exercise may be used to increase physical functions, reduce pain, and improve neuromuscular function.

Previous studies on WBV exercise for individuals with KOA focused on pain relief and physical function maintenance/improvement [12, 13]. As mentioned above, neuromuscular function is an important contributor to the development and/or progression of KOA. WBV exercise improves neuromuscular function in healthy individuals and even in patients with neurological disorders. However, limited studies have investigated the effects of WBV exercise on the neuromuscular function of individuals with KOA. Trans et al. [26] found that an 8-week WBV exercise program improves the knee extension isometric strength and threshold for the detection of passive knee extension but not the self-reported knee pain and physical function in female patients with KOA. However, they did not evaluate the physical function in daily life related to KOA (i.e., range of joint motion). The passive proprioceptive test on the knee only measured knee extension, and the neuromuscular response of the lower extremity muscle and joint biomechanics were not measured. Clearly, the effects of WBV exercise on neuromuscular function, joint biomechanics, and physical functioning in the daily life of individuals with KOA must be investigated further to understand the clinical effects and relevant mechanism of the treatment. Accordingly, the present study attempts to investigate the effects of WBV intervention on knee pain and range of joint motion and to evaluate the joint biomechanics, daily physical functions, knee and ankle proprioception, and neuromuscular response of patients with KOA. We will conduct a prospective, single-blind randomized controlled trial to investigate the efficacy of a 12-week WBV program compared with lower extremity resistance training (RT) and health education on comprehensive outcomes in individuals with KOA. The results of this study will determine the effectiveness of, and provide scientific evidence for, WBV training in individuals with KOA.

## Methods/design

### Study design

**Fig. 1:**
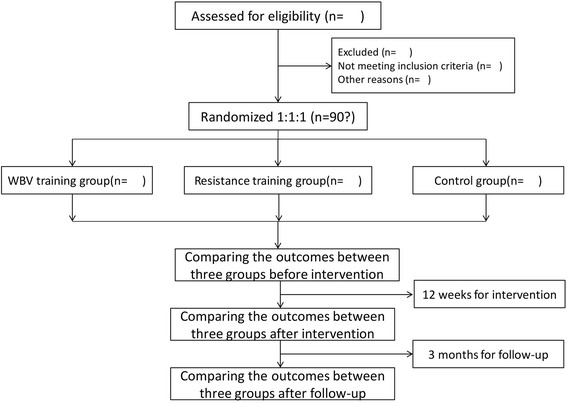
Flow diagram of study design

The study design is a prospective, single-blind randomized controlled trial. Three intervention programs, namely, a WBV exercise program, lower extremity RT, and a health education program, will be included in the study (Fig. 1). This study will be conducted at the Sport Medicine and Rehabilitation Center, Shanghai University of Sport. The power analysis with settings at *α* = 0.05, power (1 − *β*) = 0.80 and effect size = 0.25 showed that three groups of 120 participants in total was the required sample size. Given the dropouts, we decided to recruit 180 participants for the research. A total of 180 patients from community centers in Yangpu District, Shanghai, China will be included through advertisements placed in various community centers by the recruiter. A multidisciplinary team composed of clinicians, physiotherapists, and exercise specialists will run the program.

Prior to the initiation of the study, all participants will fill out the questionnaire with their details, including medical history and past and present job status. Participants will also complete the Mini-Mental State Examination and the Activities of Daily Living test, and will describe their exercise habits (frequency and duration). Informed consent will be requested from each participant prior to the inclusion in the study.

Participants who meet all study inclusion criteria and consent to participate will be randomly allocated to the WBV exercise, lower extremity RT, and health education groups via using computer-generated randomization by a research designer. The study nurse will assign participants to the interventions. Only the research designer and trainers will know the randomized assignments. The assessors will be unaware of the randomization and will not be involved in the exercise interventions. This study will include assessments at the following time points: before intervention, after 3 months of intervention, and after 3 months of further follow-up with no active intervention. The total study period will be 6 months. Additional files 1 and 2 represent SPIRIT Checklist, and schedule of enrollment, interventions and assessments﻿.

### Participants

#### Inclusion criteria

Participant selection will be based on the classification criteria of the American Rheumatism Association for KOA [27]:

1. Men or women with radiographic diagnostic criteria of definite KOA (unilateral or bilateral), with reports of pain symptoms for at least 3 months
2. Mild-to-moderate KOA (Lequesne Knee Score = 1 to 7)
3. Age 50–70 years
4. Medication not expected to change during the study period
5. Availability: three times a week over 3 months

#### Exclusion criteria

Exclusion criteria include the following:

1. Knee surgery in the past 6 months
2. Acute symptomatic OA
3. A minimum pain intensity score of more than 7 on a Visual Analog Scale (VAS)
4. Other muscular, joint, or neurological conditions that affect lower limb function
5. Unable to walk unaided
6. Currently undertaking a structured exercise program for KOA
7. Alzheimer’s disease, Parkinson’s disease, and motor neuron disorders
8. Diabetes mellitus, cardiac or respiratory insufficiency, and inability to understand the procedure

#### Withdrawal criteria and management

Withdrawal from the study will be allowed based on the following:

1. The participant makes such a request
2. The participant develops a serious disease, such as heart disease or stroke, and continuing their participation becomes inappropriate in the opinion of the investigator
3. The participant has an adverse reaction related to the WBV exercise or lower extremity RT

#### Participant recruitment

Participants will be recruited through advertisements placed in community centers and distributed to health care providers (medical practitioners, rheumatologists, and physiotherapists). Participants who satisfy the inclusion criteria will be contacted by the same investigator to confirm their willingness to take part in the trial and to arrange the baseline assessment of outcomes. Participants will provide written informed consent before the baseline assessment.

### Interventions

#### WBV exercise group

Participants in the WBV exercise group will participate in their training program 3 days per week with at least 1 day between each session for 12 weeks. WBV training will be conducted using a vibration device (AV001; BODYGREEN, Xiushui Township, Taiwan). The participants will stand with slightly bent knees (30°) on the platform and without shoes. Each session of WBV exercise will last 30 min (60 s for vibration, and 60 s for rest). The vibration parameters in the WBV training group will as follows: frequency (35–40 Hz), amplitude (4–6 mm), and acceleration ranged from (2–5 g). These parameters were considered sufficient by Delecluse for achieving the desired physiologic effects [28].

#### RT group

The RT group will undertake three training sessions per week for 12 weeks. Each session will take approximately 30 min. Each training session will be supervised by an exercise specialist to ensure that each exercise is performed using the correct method. Both lower limbs will be trained using the following exercises: knee extension, knee flexion, ankle plantarflexion, ankle dorsiflexion, extended leg raises, hip abduction, hip adduction, and hip extension. The exercise regimen will comprise three sets of 10 repetitions for each of these exercises. Each set will be performed bilaterally, starting with the less affected limb. These exercises, which have been widely used in previous studies [29, 30], will be selected primarily to strengthen the muscles directly supporting the knee. Thera-Band® resistance bands (The Hygenic Corporation, Akron, OH, USA) will be used during the RT. For participants who cannot perform 10 repetitions with the lightest resistance band for a given exercise at the baseline, the maximum number of repetitions that can be completed satisfactorily will be prescribed with an initial goal of progressing to 10 repetitions maximum. Otherwise, RT exercises will be progressed by adding a greater stretch to the prescribed band to provide greater resistance or by moving up to the next strength of resistance band.

#### Control group

The control group will attend one 60-min group session per week. The session will consist of a 30-min lecture, followed by a 30-min discussion. The control group will receive 12 weeks of health education. The lectures will cover health-related topics, such as OA, aging, and nutrition. The participants in the control group will be asked to maintain their previous lifestyle and not to take part in any other regular rehabilitation programs.

### Outcome measures

All outcome measure assessments will be performed by the main and blinded research assistant at the baseline, 3 months (after intervention), and 6 months (follow-up). A demographic questionnaire will be completed before the intervention. Demographic information includes participant characteristics (i.e., sex, age, Body Mass Index, affected side, Lequesne Knee Score, and current drug treatment). Each test will be conducted by the same assessor.

#### Primary outcome measures

##### Anthropometric measurements

The body weight and height of each participant in minimal clothing and bare feet will be measured. Body height will be measured to the nearest 0.5 cm with a fixed stadiometer (Holtain Ltd., Crymych, Dyfed, UK). Body weight will be measured to the nearest 0.1 kg on a standard scale (TBF 543 model; Tanita, Tokyo, Japan). Body Mass Index will be calculated as follows:

$$ \mathrm{Body}\  \mathrm{mass}\  \mathrm{in}\mathrm{dex}\kern0.5em =\kern0.5em \mathrm{Body}\  \mathrm{weight}\  \mathrm{in}\ \mathrm{kg}/{\left(\mathrm{Body}\  \mathrm{height}\  \mathrm{in}\ \mathrm{m}\right)}^2. $$

##### Gait analysis during walking and stair climbing

Kinematics and kinetics data will be collected and analyzed using a Vicon Motion Analysis System (Vicon MX-13, Oxford Metrics, Oxford, UK) with nine infrared cameras that record three-dimensional motion at 200 Hz and coupled with force plates (models 9286AA, Kistler Instruments Corp., Winterthur, Switzerland) that record ground reaction force at 1000 Hz. The trajectories of 45 retroreflective markers (14 mm in diameter) will be captured at different landmarks of the participant according to a plug-in gait marker set.

These biomechanical data will be collected while each participant performs three tasks:

1. Level walking at preferred speed and faster speed
2. Stair climbing

The experimental staircase consists of six steps. The step dimensions are 17.8 cm (height), 91.5 cm (wide) and 28.0 cm (tread) with a handrail [31]. Three force platforms (9286AA, Kistler Instruments Corp., Winterthur, Switzerland) will be embedded in the second, third, and fourth steps of the staircase. The participants will be asked to walk, ascend, and descend at their natural speed without any reference to the force platforms.

##### Knee extensors and flexors strength

Knee extensor and flexor strength of the affected knee joint will be measured using an isokinetic dynamometer (850, Biodex, Shirley, NY, USA). The assessor will secure the participants to the device at the upper chest, pelvis, and distal femur on the tested side with Velcro straps. Three maximum concentric contractions for the knee extensors and flexors will be performed at an angular velocity of 90°/s. All data will be normalized by kilogram of body weight. The highest peak torque will be used for analysis. The dynamic endurance of the knee extensors and flexors will be assessed by measuring 40 repeated maximum isokinetic contractions at an angular velocity of 180°/s. The work of each contraction in the knee moving angle of 80°–10° will be recorded. The Endurance Index is defined as the ratio of the work performed during the last five contractions over the first five contractions.

##### Proprioception test of the knee and ankle

This measurement method was reported in our previous studies [32]. Knee and ankle proprioception will be tested using an electrically driven movable frame. During the tests, the participant will sit on a chair with the dominant leg supported by the frame. The leg can be passively moved in a flexed or extended direction at a velocity of 0.4°/s. Once the participant is able to detect the leg motion, they will press a handheld “stop” button and confirm the direction of the motion. The rotation angles of the frame will be determined as the threshold for the detection of the knee and ankle joint. The mean values of the three trials in one direction will be used for the analysis.

##### Neuromuscular response

Neuromuscular response, indicated by muscle latency, will be assessed using electromyography (EMG) of the leg muscles while an unexpected perturbation is applied to the ankle. A customized trapdoor with an 18° tilt angle will be used to generate an ankle inversion perturbation while the participants stand barefoot on the trap doors. The Noraxon EMG system (Noraxon USA Inc., Scottsdale, AZ, USA) will be used to collect surface EMG signals from five muscles (rectus femoris, semitendinosus, gastrocnemius, peroneus longus, and anterior tibialis) of the right leg and onset signals at the trapdoor tilting with a sampling frequency of 1000 Hz. Both feet will be randomly tilted at least seven times to decrease anticipatory effects. The onset latency of the muscles refers to the time interval in milliseconds (ms) between the trapdoor initiation and the first rising front of the EMG burst from the baseline. The EMG onset will be visually determined by an experienced researcher.

Furthermore, muscle activation will be determined during level walking and stair climbing. EMG data will be collected from eight lower extremity muscles in accordance with the recommended muscles for gait analysis from Winter and Yack [33]; these muscles are the ipsilateral and contralateral erector spinae at the level of the iliac crest, rectus femoris, vastus medialis, vastus medialis, tibialis anterior, biceps femoris, peroneus longus, and gastrocnemius. The Noraxon EMG system (Noraxon USA Inc., Scottsdale, AZ, USA) will be used to collect surface EMG signals from these muscles of the right leg with a sampling frequency of 1000 Hz. The EMG signals from five complete gait cycles per task will be used in data reduction. Prior to the EMG data collection, maximal isometric contraction data will be gathered for each muscle. The mean EMG amplitude and on-off muscle timing during a gait cycle will be used in the data analysis [34].

#### Secondary outcome measures

##### Self-reported pain and physical functional capacity

A 10-cm Visual Analog Scale (VAS; 0, no pain; 10, maximal pain) will be used to assess self-reported pain related to knee joint movement.

The Western Ontario and McMaster Universities Osteoarthritis Index (WOMAC), a validated [35] and widely used OA-specific, self-reported questionnaire that assesses pain, stiffness, and function in patients with OA of the knee will be used to assess the symptoms and function of the participants. The WOMAC includes three subscales (pain, stiffness, and physical function) and 24 questions. Each question is scored from 0 to 4, with 0 indicating no pain and 4 indicating great pain.

The 36-item Short Form Health Survey (SF-36), which is a large-scale measurement tool used in evaluating the positive and negative aspects of the overall health status, will be used to indicate the overall health status of the participants. The parameter scores range from 0 and 100, and higher values indicate better health status [36]. This tool is easy to use, acceptable to patients, and fulfills the stringent criteria of reliability and validity [37].

The Beck Depression Inventory, a common scale used to assess the severity of depression of individuals, will be used to determine whether the WBV exercise and control group programs have different psychological effects on the participants [38]. Higher scores indicate high levels of depression. The achievement of more functional benefits with the WBV training program than with the control group programs might have psychological reflections.

##### Physical performance measures

The Berg Balance Scale is a widely used clinical test of the static and dynamic balance abilities of a person, and it comprises a set of 14 simple, balance-related tasks, ranging from standing up from a sitting position to standing on one foot. The total score ranges from 0 to 56, with 0–20 corresponding to a high fall risk, 21–40 a medium fall risk, and 41–56 a low fall risk [39].

The Timed Up and Go test (TUG), a reliable and valid measure of balance and mobility in patients with KOA [40], will be used to assess functional performance. Participants will be asked to stand up from a seat, walk 3 m, turn around, walk back, and sit down again. The test will be performed twice, and the faster time will be recorded. The entire walking test will be timed using a chronograph (in seconds).

The 6-minute Walk Distance test (6MWD), which is a simple method to indicate functional performance reliably and is frequently used in OA-related trials, will be used to measure the walking function of the participants. The distance walked in 6 min will be recorded in meters [41].

### Statistical analysis

SPSS for Windows, version 17.0 (SPSS Inc., Chicago, IL, USA) will be used for statistical analysis. If any participant withdraws from the trial, the missing values will be replaced by the last assessment score of the participant. And all available data will be analyzed using an intention-to-treat analysis. Chi-squared tests will be used to test for demographic differences among the WBV training, RT, and control groups.

An intention-to-treat analysis will be performed by including all participants in the analysis according to the original group allocation. The follow-up will be maximized regardless of the program attendance. Repeated-measurement analysis of variance will be used to evaluate for between- and within-group difference. Bonferroni’s post hoc test will used for comparing the results. Data will be presented as the mean and standard deviation, and significance will be set at 0.05.

## Discussion

Various exercises have been recommended as potential treatments for KOA [42–45]. Given its feasibility and safety, WBV exercise has been recommended as an alternative treatment to improve the function and self-reported disease status of individuals with KOA [12, 13, 46]. Furthermore, one study found no progression in the self-reported knee pain of a patient with KOA after 8 weeks of WBV training [26]. However, several studies failed to find significant improvements in pain intensity and functional performance [47, 48]. Recently, several systematic reviews and meta-analyses investigated the therapeutic effects of WBV exercise in individuals with KOA [12, 13, 49]. In general, evidence supports the notion that WBV exercise reduces pain and improves physical functions in individuals with KOA [12, 13]. Furthermore, no significant difference was found in the pain intensities and self-reported status of patients who performed WBV exercise and other exercises [49]. Currently, limited evidence is available to support the effect of WBV on patients with KOA. Considering the lack of data-appropriate RCTs, the American College of Rheumatology did not recommend WBV exercise as a treatment for KOA [9]. Therefore, well-designed RCTs are required to determine the therapeutic effect of WBV exercise on individuals with KOA.

As mentioned above, optimal neuromuscular function contributes to the development and/or progression of KOA [18]. Individuals with KOA have deficiencies in the detection of joint position [50–52]. WBV exercise improves muscle strength, power, joint proprioception, balance, and flexibility in sedentary and older individuals [23]. It also improves functional performance, such as walking, and postural stability, of individuals with KOA. Indeed, previous studies have demonstrated that WBV may be used as an efficient and alternative option for improving muscle strength of the lower extremity and joint proprioception [26, 48, 53, 54]. To date, no RCT study has been designed to investigate the effects of WBV exercise on neuromuscular control in individuals with KOA.

The strengths of our protocol are as follows: (1) investigation responses on neuromuscular function use WBV exercise and RT in individuals with KOA, which has not been described in this population; (2) the relatively long study duration, with an intervention period of 3 months and a follow-up period (with no active intervention) of 3 months, for a total study period of 6 months; (3) extensive follow-up to monitor the effects of WBV exercise and RT on the physical performance and neuromuscular function of individuals with KOA; (4) measurement of neuromuscular function provides advanced findings to explain the possible mechanisms of functional improvement in individuals with KOA; and (5) a comprehensive dissemination plan to ensure the adequate uptake of knowledge generated in this study. However, this study also has several limitations. First, recruitment is limited to individuals with mild-to-moderate KOA; thus, the study results may only be valid for individuals with mild-to-moderate KOA. The use of a large sample size will also address the current study’s limitation of relying on a relatively small study population and the fact that it is not a multicenter trial.

In conclusion, this study attempts to estimate the effect of WBV intervention on outcomes, including daily life function and neuromuscular control, in individuals with KOA. The study results may provide evidence to support the beneficial effects of a WBV exercise program on the physical performance and neuromuscular control of individuals with KOA. The findings of this study will fill the research gap in the efficacy of WBV based on the results of the proposed project. Further comprehensive research on the exercise rehabilitation of KOA will be proposed. Furthermore, the possible mechanism of postural instability in KOA patients may be discussed.

### Trial status

Participant recruitment is ongoing.

## Additional file

Additional file 1: SPIRIT Checklist. (DOC 121 kb)
Additional file 2: Schedule of enrollment, interventions, and assessments. (PNG 401 kb)

## Abbreviations

6MWD: 6-minute Walk Distance
BMI: Body Mass Index
EMG: Electromyography
KOA: Knee osteoarthritis
OA: Osteoarthritis
RT: Resistance training
TUG: The Timed Up and Go test
VAS: Visual Analog Scale
WBV: Whole body vibration
WOMAC: The Western Ontario and McMaster Universities Osteoarthritis Index
