# Supplementary figures and images for: Effects of whole body vibration exercise on neuromuscular function for individuals with knee osteoarthritis: study protocol for a randomized controlled trial
Source: Trials. 2017 Sep 20;18:437. doi: 10.1186/s13063-017-2170-6 (PMC5607567; doi:10.1186/s13063-017-2170-6)

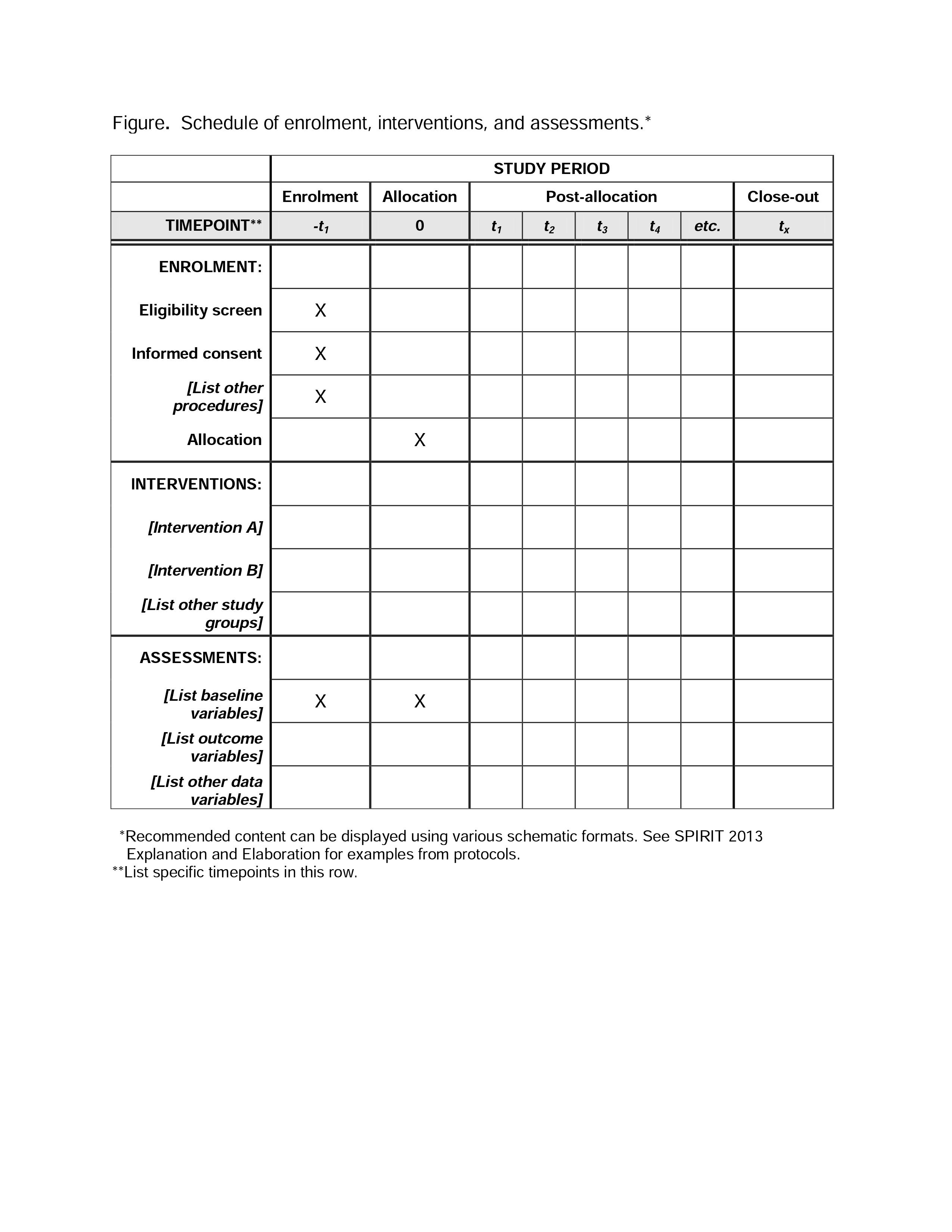

Supplement: Additional file 2: — Schedule of enrollment, interventions, and assessments. (PNG 401 kb) [file 13063_2017_2170_MOESM2_ESM.png]
